# Polyunsaturated Branched-Chain Fatty Acid Geranylgeranoic Acid Induces Unfolded Protein Response in Human Hepatoma Cells

**DOI:** 10.1371/journal.pone.0132761

**Published:** 2015-07-17

**Authors:** Chieko Iwao, Yoshihiro Shidoji

**Affiliations:** Molecular and Cellular Biology, Graduate School of Human Health Science, University of Nagasaki, Nagasaki, Japan; University of Hong Kong, HONG KONG

## Abstract

The acyclic diterpenoid acid geranylgeranoic acid (GGA) has been reported to induce autophagic cell death in several human hepatoma-derived cell lines; however, the molecular mechanism for this remains unknown. In the present study, several diterpenoids were examined for ability to induce *XBP1* splicing and/or lipotoxicity for human hepatoma cell lines. Here we show that three groups of diterpenoids emerged: 1) GGA, 2,3-dihydro GGA and 9-*cis* retinoic acid induce cell death and *XBP1* splicing; 2) all-*trans* retinoic acid induces *XBP1* splicing but little cell death; and 3) phytanic acid, phytenic acid and geranylgeraniol induce neither cell death nor *XBP1* splicing. GGA-induced ER stress/ unfolded protein response (UPR) and its lipotoxicity were both blocked by co-treatment with oleic acid. The blocking activity of oleic acid for GGA-induced *XBP1* splicing was not attenuated by methylation of oleic acid. These findings strongly suggest that GGA at micromolar concentrations induces the so-called lipid-induced ER stress response/UPR, which is oleate-suppressive, and shows its lipotoxicity in human hepatoma cells.

## Introduction

Geranylgeranoic acid (GGA) is a natural diterpenoid found in several medicinal herbs including turmeric [1]. GGA and its derivatives have been repeatedly reported to induce cell death in human hepatoma cells [2,3]. In placebo-controlled randomized clinical trials, 4,5-didehydroGGA both safely and effectively prevented tumor recurrence in postoperative hepatoma-free patients [4–6]. While 4,5-didehydroGGA has been utilized for clinical trials, it has so far not been identified in natural resources. As both 4,5-didehydroGGA and naturally occurring GGA are able to induce cell death in human hepatoma-derived HuH-7 cells [1], we investigated the mechanisms by which natural GGA induces cell death in human hepatoma cells.

Over the past 15 years, we have reported various cell-death related effects of GGA at micromolar concentrations in several cell culture systems: loss of mitochondrial membrane potential in HuH-7 cells [3], hyper-production of superoxide in transformed fibroblastic 104C1 cells [7], and rapid downregulation of cyclin D1 in three human hepatoma-derived cell lines [8]. We recently identified that HuH-7 cells undergo an incomplete autophagic response following GGA treatment [9], which may contribute to GGA-induced cell death. Here, while the initial phase of autophagy occurs, the maturation of autolysosomes or later stages of autophagy fail to proceed, leading to substantial accumulation of early/initial autophagic vacuoles, LC3β-II, and p62/SQSTM in HuH-7 cells [9]. The molecular mechanisms underlying why the later stages of autophagy and the fusion of autophagosomes with lysosomes fail remain to be elucidated, however, and in the present study we focus on what kind of cellular events GGA initially induces during incomplete autophagic response and the mechanisms whereby this is linked to cell death.

Possible mechanisms by which GGA could induce autophagy include impairing mTOR- mediated suppression of autophagy through starvation stress such as amino acid depletion [10], activation of autophagy-initiating gene product, ATG4, which is induced by oxidative stress [11], and the endoplasmic reticulum (ER) stress-mediated unfolded protein response (UPR) as an upstream signal from lipotoxicity with fatty acids [12]. Of these three potential triggering mechanisms, we focused on UPR-mediated induction of autophagy, as our previous data identified that GGA induced rapid translational downregulation of cyclin D1 [8]. This strongly suggests upregulation of the protein kinase R (PKR)-like endoplasmic reticulum kinase (PERK) pathway, which is one branch of the mammalian UPR and is associated with blocking of cyclin D1 translation [13].

Here, we describe the induction of an ER stress-triggered UPR involving *XBP1* mRNA splicing and the upregulation of PERK- downstream gene expression by GGA treatment, resulting in the initiation of autophagy and cell death. Furthermore, we demonstrate that several acyclic diterpenoid acids are capable of inducing cell death in human hepatoma cells.

## Materials and Methods

### Materials

GGA, phytenic acid, farnesoic acid and geranoic acid were generous gifts from Kuraray (Okayama, Japan). All-*trans* retinoic acid (ATRA) and 9-*cis* retinoic acid (9CRA) were obtained from Wako, Osaka, Japan. (*S*)-2,3-dihydroGGA, (*R*)-2,3-dihydroGGA and dolichoic acid were synthesized and provided by Dr. Sagami of Tohoku University, Sendai, Japan. Geranylgeraniol (GGOH), phytanic acid, arachidic acid, palmitic acid, oleic acid, fatty acid-free bovine serum albumin (BSA) and tunicamycin were purchased from Sigma Aldrich, St. Louis, MO, USA. IRE1 inhibitor III, 4μ8C (#412512), was from Calbiochem, Merck Millipore Japan, Tokyo, Japan.

### Cell culture

Human hepatoma-derived HuH-7, HepG2 and PLC/PRF/5 cells were obtained from RIKEN BioResource Center, Tsukuba, Japan, and cultured in high-glucose Dulbecco’s modified Eagle’s medium (DMEM; Wako) supplemented with 5% fetal bovine serum (FBS; Hyclone Laboratories, Thermo Fisher Scientific, Waltham, MA, USA). The Hep3B cells were obtained from DS Pharma Biomedical, Osaka, Japan, and maintained in DMEM containing 10% FBS and MEM nonessential amino acid solution (Sigma Aldrich).

Cells were cultured with DMEM containing 5% or 10% FBS for 2 days followed by replacement with FBS-free DMEM for a further 2 days before drug treatment. GGA, other diterpenoids or other lipids (in ethanol) were dispersed in FBS-free medium, except that palmitic acid was dissolved in FBS-free medium containing 1% BSA at a final concentration of 400 μM and preincubated at 37°C for 1 h prior to treatment.

### Reverse-transcription real-time polymerase chain reaction (RT-qPCR)

Cells were treated with GGA or other compounds, and total RNA was isolated using the QuickGene RNA cultured Cell kit S (Wako) with QuickGene-810 (Kurabo, Osaka, Japan). cDNA was generated using the Transcriptor First Strand cDNA Synthesis kit with random hexamers (Roche Diagnostics, Basel, Switzerland). Nucleotide sequences of the PCR primers for the *DDIT3* (DNA- damage-inducible transcript 3, also known as *CHOP*), *PDIA4* (protein disulfide isomerase family A, member 4), *ACSL3* (acyl-CoA synthetase long-chain family member 3) and *28S rRNA* cDNAs are listed in S1 Table in S1 File. Real-time PCR was performed with DyNAmo Capillary SYBR Green qPCR Master mix (Finnzymes, Espoo, Finland), and cDNA on LightCycler1.5 (Roche Diagnostics) under conditions described in S2-S7 Tables in S1 File.

### Immunoblotting

Following treatment with GGA or tunicamycin, HuH-7 cells were lysed with cell lysis buffer (Tris-based buffered saline containing 1% Nonidet P40, 0.5% sodium deoxycholate and 0.1% SDS) containing complete mini protease inhibitor cocktail (Roche Diagnostics) and proteins were quantified by Bradford assay (Bio-Rad, Hercules, CA, USA). Equal amounts (15 or 30 μg) of protein per sample were separated by SDS-PAGE. The semi-dry blotted polyvinylidene fluoride membranes (Bio-Rad) were probed with rabbit polyclonal antibody against XBP1s (#619502, BioLegend, San Diego, CA, USA), LC3 (PM036, Medical Biological Laboratories, Nagoya, Japan) and β-tubulin III (T2200, Sigma Aldrich). Horseradish peroxidase (HRP)-labeled secondary antibody (GE Healthcare, Tokyo, Japan) was detected with Immobilon Western Chemiluminescent HRP substrate (Merck Millipore Japan) using an ImageQuant LAS 4000 (GE Healthcare).

### Immunofluorescence

Following treatment with GGA or other compounds, HuH-7 cells grown on glass inserts in a 24-well plate were rinsed with Ca-free PBS (PBS(-); Sigma Aldrich) and fixed for 40 min with 4% paraformaldehyde containing 2% sucrose in PBS(-), and then rinsed with PBS(-). Cells were then permeabilized with 0.5% Triton X-100 and nonspecific binding blocked with 10% FBS. Next, cells were incubated at 4°C overnight with polyclonal anti-XBP1s antibody, followed by 2.5 h incubation with Alexa-488-labeled goat anti-rabbit IgG antibody (Invitrogen, Molecular Probes, Tokyo, Japan). After rinsing with PBS(-), cells were mounted in PermaFluor (Beckman Coulter, Brea, CA, USA), covered on a glass slide, and observed under a confocal laser-scanning fluorescence microscope, LSM700 2Ch URGB equipped with Axio Observer Z1 Bio (Carl Zeiss, Göttingen, Germany).

### Live-cell imaging

HuH7/GFP–LC3 cells [9] were cultured on glass-bottomed dishes (Matsunami) at a density of 3×10^4^ cells/dish in a chamber unit (INUG2-ZIL; Tokai Hit, Hamamatsu, Shizuoka, Japan), equipped with a Carl Zeiss LSM 700 inverted laser-scanning confocal fluorescence microscope. Live-cell images were scanned after the addition of 10 μM GGA for 8 h in the absence or presence of 25 μM oleate.

### Cell viability assay

Cells (1000 cells/well) were seeded into a 96-well plate and cultured with DMEM containing 5% FBS for 2 days before replacing the medium with FBS-free DMEM for a further 2 days prior to 24 h treatments with GGA or other compounds. The CellTiter-Glo (Promega KK, Tokyo, Japan) was then used to measure cell viability by ATP levels as per manufacturer’s instructions. The luminescence was recorded by using Centro XS3 LB960 (Berthold Technologies, Wildbad, Germany). Alternatively, HuH-7 cells were seeded into 3-cm dish at a density of 3×10^4^ cells/dish and cultured with DMEM containing 5% FBS for 2 days before replacing the medium with FBS-free DMEM for a further 2 days prior to 24 h treatments with palmitic acid and oleic acid. A number of viable cells were counted by trypan blue method after detachment with trypsin treatment.

### Statistical analyses

Unless otherwise stated, where applicable results are presented as means ± standard deviation (SD) for each series of experiments with Student’s t-test was used to determine statistical significance of the difference in means between the two groups; significance was set at *: p < 0.05, **: p < 0.01 and ***: p < 0.001. For other cases, one-way or two-way ANOVA followed by Dunnett's post hoc multiple comparison tests was performed with GraphPad Prism 6 (GraphPad Software, La Jolla, CA); significance was set at *: p < 0.05, **: p < 0.01 and ***: p < 0.001.

## Results

### Rapid induction of *XBP1* mRNA splicing by GGA treatment in HuH-7 cells

**Fig 1:**
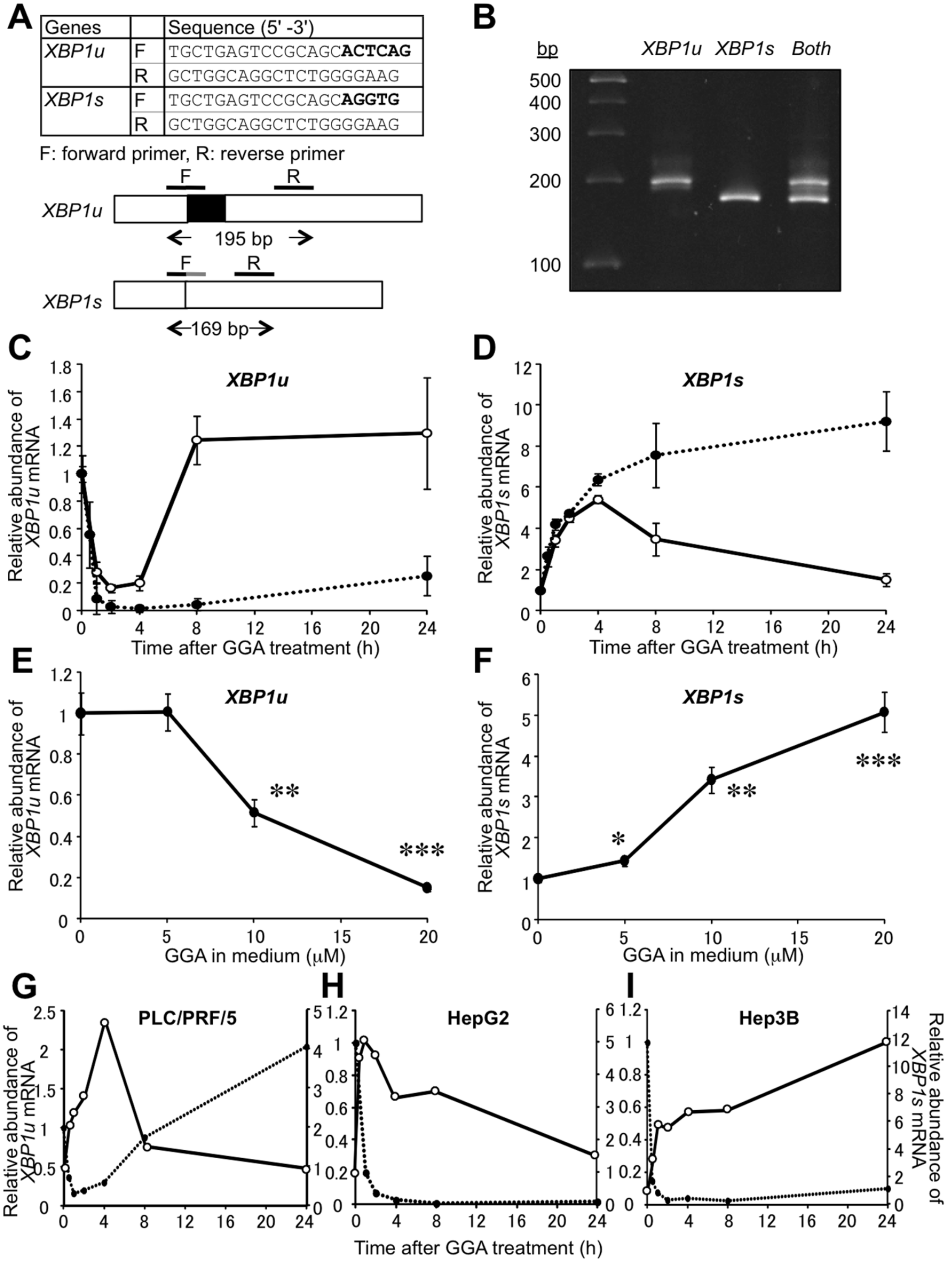
Rapid induction of *XBP1* mRNA splicing by GGA treatment in HuH-7 cells. Quantitative RT-PCR primers discriminating the spliced and unspliced forms of *XBP1* mRNA were designed and their nucleotide sequences are shown in **A**. Total mRNA was extracted from HuH-7 cells and RT-qPCR was performed using either these *XBP1u* or *XBP1s* primers. Following qPCR (45 cycles), products of *XBP1u* or *XBP1s* amplification were diluted by 10 or 50 fold, respectively, prior to electrophoresis by E-Gel 48 (4% agarose) to validate the discriminating primers (**B**). HuH-7 cells were treated with 10 (open circle) or 20 μM GGA (closed circle) for 0, 1, 2, 4, 8, and 24 h. Total mRNA was extracted to measure the cellular levels of *XBP1u* (**C**) and *XBP1s* (**D**) mRNA by quantitative reverse-transcription (RT)-PCR in duplicate. Each point represents the mean ± SD (n = 3). HuH-7 cells were treated with GGA (0–20 μM) for 1 h, and total mRNA was extracted to estimate the cellular levels of *XBP1u* (**E**) and *XBP1s* (**F**) mRNA by RT-qPCR. Each point represents the mean ± SE of seven independent experiments. Asterisks (*, **, ***) indicate statistically significant difference from a control sample at 0 h with p value of < 0.05, 0.01, 0.001, respectively, as determined by ANOVA followed by post hoc multiple comparison test. PLC/PRF/5 (**G**), HepG2 (**H**) or Hep3B (**I**) cells were treated with 20 μM GGA for 0, 0.5, 1, 2, 4, 8, and 24 h, and total mRNA was extracted to analyze XBP1u and XBP1s mRNA expression by RT-qPCR. Closed and open circles indicate the relative abundance of XBP1u and XBP1s mRNA, respectively.

First of all, we designed 2 sets of primers for real-time RT-PCR as shown in Fig 1A, in order to quantitatively and separately measure the cellular contents of unspliced *XBP1* and spliced *XBP1* mRNAs. These primer sets enabled us to completely discriminate the spliced and unspliced forms of *XBP1* mRNA after 45 cycles of PCR (Fig 1B), so that kinetics of GGA-induced splicing of *XBP1* mRNA was studied to separately measure the cellular levels of *XBP1* mRNA each forms by quantitative RT-PCR with these primer sets.

Treatment with GGA rapidly induced *XBP1* mRNA splicing in HuH-7 cells (Fig 1C and 1D). Following addition of 10 μM GGA to the culture medium, the level of unspliced *XBP1* (*XBP1u*) mRNA was immediately downregulated in a time dependent manner. Less than one-fifth the initial level of *XBP1u* mRNA was present 2 h after GGA treatment, and this fall was maintained until 4 h. However, by 8 h, the *XBP1u* level had recovered to that of control cells (bold line in Fig 1C). Accordingly, the level of spliced *XBP1* (*XBP1s*) mRNA was increased 3.4-fold at 1 h and up to 5.4-fold at 4 h compared with the control level. From 8–24 h, the cellular levels of *XBP1s* mRNA gradually decreased from 3.5- to 1.5-fold (bold line in Fig 1D), suggesting that the *XBP1* splicing induced by treatment with 10 μM GGA was a transient response.

Conversely, the effect of treatment with 20 μM GGA on *XBP1* splicing appeared sustainable during the experiment. Here, a more potent decrease in *XBP1u* mRNA was observed, with less than one-twentieth of the initial amount remaining 2 h following treatment, and one-fiftieth at 4 h. This decrease was sustained at 24 h (dotted line in Fig 1C). Treatment with 20 μM GGA upregulated the level of *XBP1s* mRNA at 4 h in a similar manner to 10 μM GGA treatment; however, this upregulation continued and by 24 h the *XBP1s* mRNA level had increased 9.2-fold over that of control (dotted line in Fig 1D). Two-way ANOVA revealed that kinetics of the cellular levels of either *XBP1u* or *XBP1s* mRNA was significantly different between 10 and 20 μM GGA-treated cells.

We next examined whether GGA-induced *XBP1* splicing was concentration-dependent by measuring *XBP1u* levels at 1 h following treatment with 0–20 μM GGA. No change in *XBP1u* level was observed following administration of 5 μM GGA (Fig 1E), although a slight but significant increase in the level of *XBP1s* mRNA was detected (Fig 1F). Treatment with 10 μM GGA significantly decreased the *XBP1u* mRNA level while 20 μM GGA almost completely depleted all *XBP1u* mRNA, giving an apparent EC_50_ of 10 μM (Fig 1E). Furthermore, GGA increased *XBP1s* mRNA levels in a dose-dependent manner from a 1.4-fold increase over control at 5 μM to a 5.1-fold increase at 20 μM (Fig 1F).

A time-dependent induction of *XBP1* mRNA splicing also followed treatment with 20 μM GGA in three other human hepatoma cell lines: PLC/PRF/5 (Fig 1G), HepG-2 (Fig 1H) and Hep3B (Fig 1I).

### Structure specificity of diterpenoid-induced splicing of *XBP1* mRNA in HuH-7 cells

**Fig 2:**
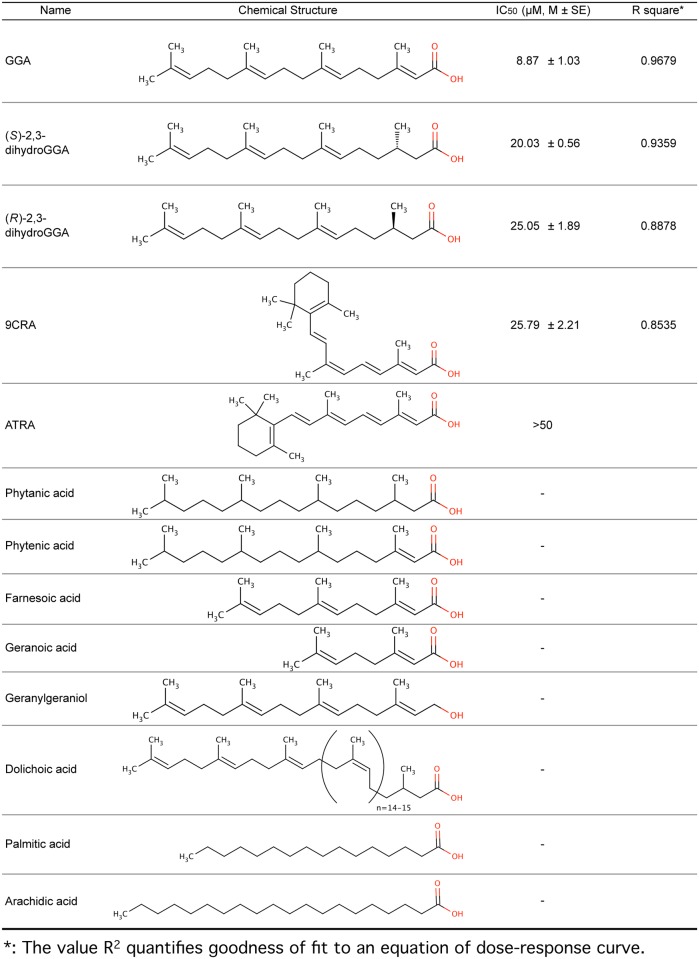
List of compounds tested and their IC_50_ values determined by CellTiter Glo assay and GraphPad Prism.

**Fig 3:**
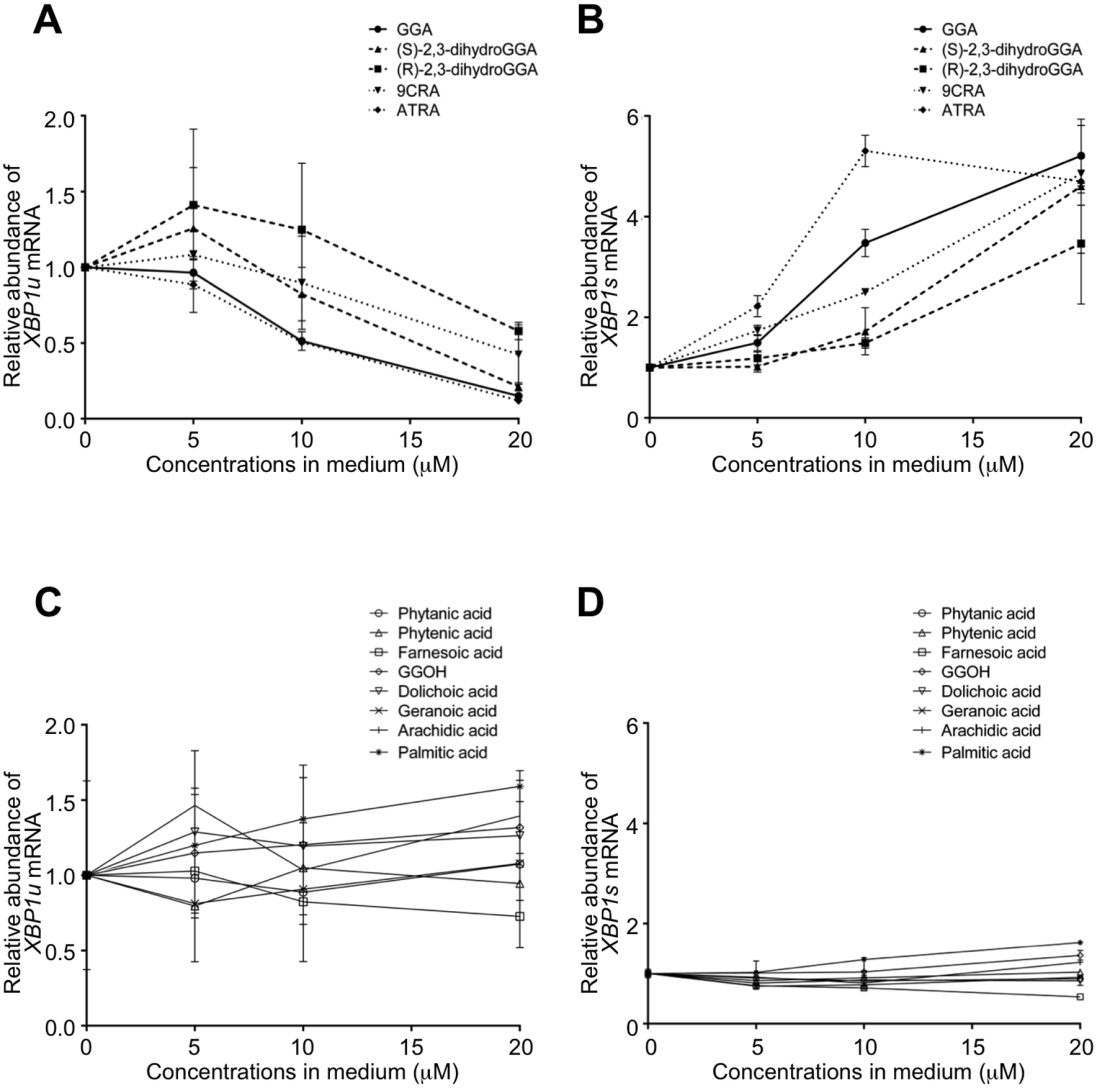
Specificity of diterpenoid-induced splicing of *XBP1* mRNA in HuH-7 cells. HuH-7 cells were treated with GGA, (*S*)-2,3-dihydroGGA, (*R*)-2,3-dihydroGGA, ATRA, 9CRA, phytanic acid, phytenic acid, farnesoic acid, GGOH, dolichoic acid, geranoic acid, palmitic acid, or arachidic acid (0–20 μM) for 1 h. Total mRNA was extracted to analyze the cellular levels of *XBP1u* (**A, C**) and *XBP1s* (**B, D**) mRNA by RT-qPCR. Panels **A, B**; GGA, (*S*)-2,3-dihydroGGA, (*R*)-2,3-dihydroGGA, ATRA, or 9CRA are shown. These acids significantly changed both mRNA levels as revealed by ANOVA (p < 0.05). Panels **C, D**; phytanic acid, phytenic acid, farnesoic acid, GGOH, dolichoic acid, geranoic acid, palmitic acid and arachidic acid (0–20 μM) are plotted. Effects of each compound in this group on both mRNA levels are not statistically significant. Each point represents the mean ± SE (n = 1–7).

To determine whether GGA- analogous compounds could also induce *XBP1* mRNA splicing, we treated HuH-7 cells with (*S*)-2,3-dihydroGGA, *(R*)-2,3-dihydroGGA, ATRA, 9CRA, phytanic acid, phytenic acid, farnesoic acid, GGOH, dolichoic acid, geranoic acid, palmitic acid, and arachidic acid (chemical structures depicted in Fig 2). Fig 3A shows the dose-dependent effects of these compounds on *XBP1u* mRNA levels in HuH-7 cells after 1 h treatment. ATRA decreased *XBP1u* mRNA level in a concentration-dependent manner essentially identical to GGA. 9CRA, a stereoisomer of ATRA, and (*S*)-2,3-dihydroGGA also both induced splicing of *XBP1u* mRNA, but to a lesser degree than GGA. (*R*)-2,3-dihydroGGA, an enantiomer of (*S*)-2,3-dihydroGGA, triggered a decrease in *XBP1u* mRNA only at 20 μM. Other compounds listed in Fig 2 exhibited no detectable effect on *XBP1u* mRNA levels (see Fig 3C).

Fig 3B depicts the concentration- dependent appearance of *XBP1s* mRNA 1 h following treatment with the aforementioned compounds. ATRA induced an upregulation of *XBP1s* mRNA, with saturation at 10 μM. In accordance with the changes of *XBP1u* mRNA, 9CRA was a weaker inducer of *XBP1s* mRNA than ATRA and GGA, as were both (*S*)- and (*R*)-2,3-dihydroGGA. From most to least potent, the *XBP1*-splicing inducing activity of these diterpenoid acids was: ATRA > GGA > 9CRA > (*S*)-2,3-dihydroGGA > (*R*)-2,3-dihydroGGA. The other compounds used failed to induce any change to the *XBP1s* mRNA level (Fig 3D).

### Nuclear accumulation of XBP1s after GGA treatment

**Fig 4:**
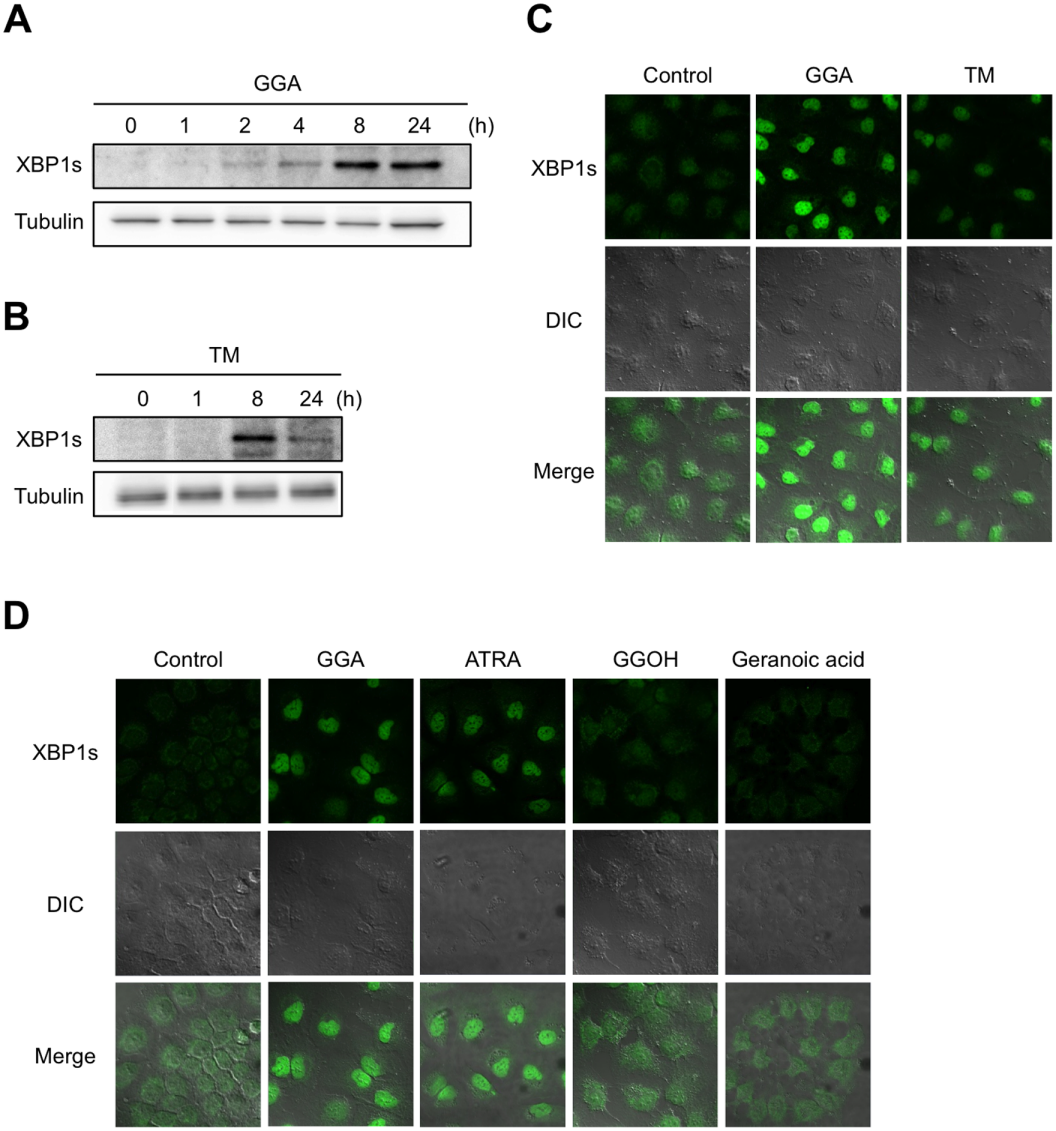
Nuclear accumulation of XBP1s after GGA treatment. **A, B**: HuH-7 cells were treated with 20 μM GGA (**GGA**) for 0, 1, 2, 4, 8, and 24 h or 0.25 μg/mL tunicamycin (**TM**) for 0, 1, 8, and 24 h. Whole-cell lysates were prepared, 30 μg of total protein per lane were used for GGA-treated cell lysates, 15 μg were used for TM-treated cell lysates and XBP1s levels were analyzed by western blotting. Total tubulin-βIII was used as a loading control. **C**: HuH-7 cells were cultured under the following conditions: vehicle control (**Control**), 20 μM GGA for 8 h (**GGA**) or 0.25 μg/mL tunicamycin for 8 h (**TM**). Green fluorescence indicates the distribution of XBP1s. DIC: differential interference contrast. **D**: HuH-7 cells were cultured with vehicle alone (**Control**), 10 μM GGA (**GGA**), 10 μM ATRA (**ATRA**), 10 μM GGOH (**GGOH**) or 10 μM geranoic acid (**Geranoic acid**) for 8 h. Green fluorescence indicates the distribution of XBP1s protein in cells.

Treatment with 20 μM GGA led to detection of XBP1s protein at as early as 2 h and the protein became evident at 8 h and was still found until 24 h following treatment (Fig 4A). Tunicamycin induced a transient increase of the cellular XBP1s protein level at 8 h and the protein level was decreased at 24 h after the treatment (Fig 4B).

We then examined the subcellular distribution of GGA-induced XBP1s protein in HuH-7 cells, because XBP1s acts as a transcription factor that migrates to the nucleus. Fig 4C depicts the distribution of XBP1s protein in both the cytoplasmic and nuclear space of HuH-7 cells under ethanol-control condition. At 8 h after addition of GGA, however, the XBP1s protein signal was increased and mainly localized in the nuclear space. Treatment with tunicamycin produced similar changes in subcellular distribution of XBP1s (Fig 4C).

Fig 4D shows that 2 isoprenoids with no activity to induce UPR such as GGOH and geranoic acid failed to translocate XBP1s to nuclei, whereas other UPR-inducing isoprenoids of GGA and ATRA were both effective to induce nuclear translocation of XBP1s.

### Specificity of diterpenoid-induced cell death in HuH-7 cells

**Fig 5:**
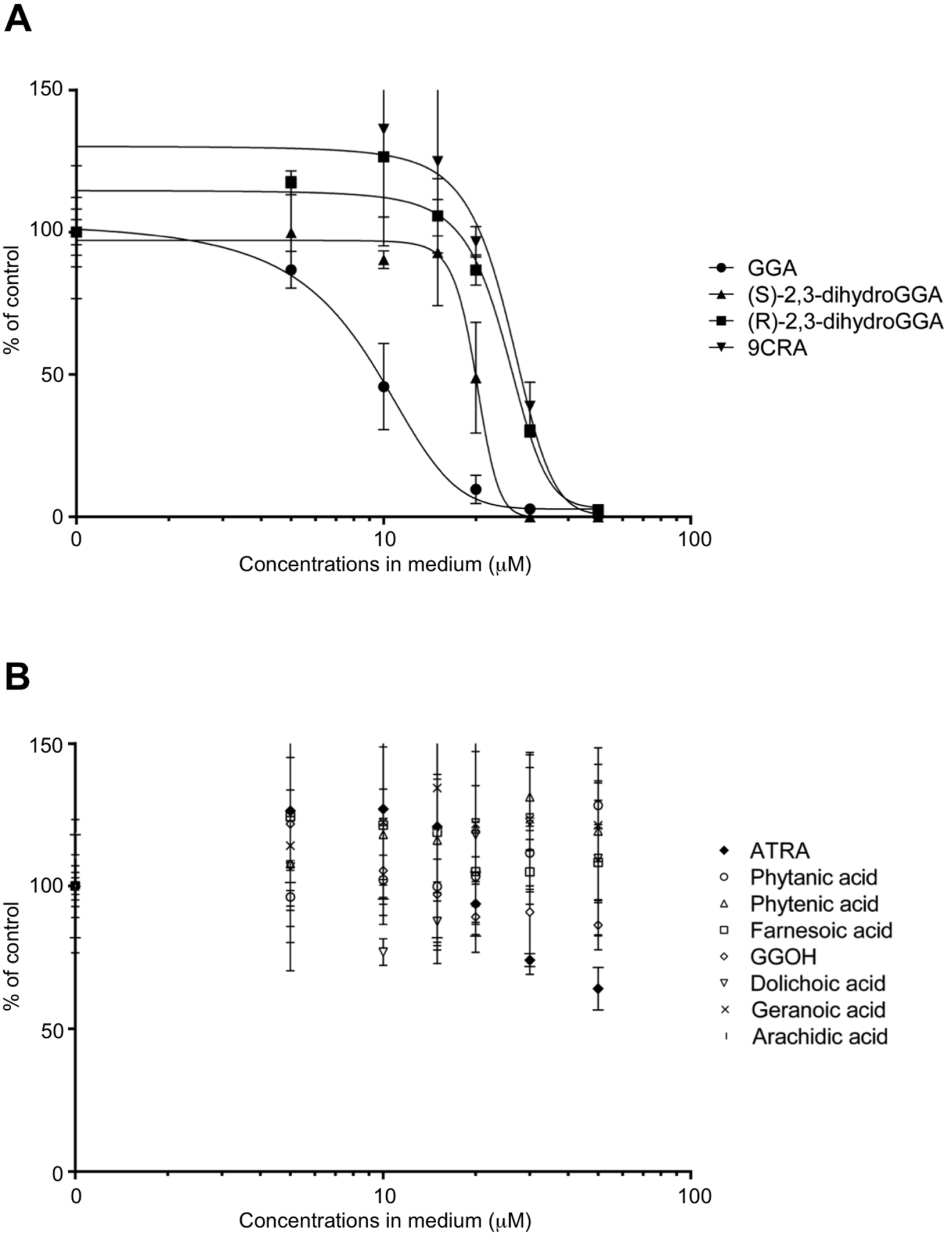
Specificity of diterpenoid-induced cell death in HuH-7 cells. Viable cells were measured using the CellTiter-Glo assay at 24 h after treatment with 0–50 μM of GGA, (*S*)-2,3-dihydroGGA, (*R*)-2,3-dihydroGGA, or 9CRA (**A**), and ATRA, phytanic acid, phytenic acid, farnesoic acid, GGOH, dolichoic acid, geranoic acid, or arachidic acid (**B**). Experiments were performed in triplicate. Values are the means ± SE (n = 3). Inhibition curves for each compound in panel **A** were created to find IC_50_ (shown in Fig 2) using GraphPad Prism 6, whereas the software failed to fit a dose-response curve to find IC_50_ for each compound in panel **B**.

To examine whether GGA-induced *XBP1* splicing is connected to GGA-induced cell death, we conducted cell viability assays following similar treatments of HuH-7 cells to those described above. The degree of cell viability at 24 h following treatment with 0–50 μM of the various compounds was assessed using the CellTiter-Glo assay. The three acyclic diterpenoid acids GGA, (*S*)-2,3-dihydroGGA, and (*R*)-2,3-dihydroGGA, which all triggered *XBP1* mRNA splicing, induced cell death in a dose-dependent manner (Fig 4A). The potency of this cell death-inducing activity was: GGA > (*S*)-2,3-dihydroGGA > (*R*)-2,3-dihydro GGA, as judged by calculating the IC_50_ values shown in Fig 2. Although both ATRA and 9CRA were capable of inducing *XBP1* mRNA splicing, 50 μM ATRA treatment still resulted in 64% cell viability, so an IC_50_ could not be determined. In contrast, 9CRA demonstrated an IC_50_ of 26 μM, which is slightly greater than the IC_50_ of (*R*)-2,3-dihydroGGA. All other compounds failed to induce cell death, as shown in Fig 5B and Fig 2.

### GGA-induced XBP1 splicing shared a similarity with lipid-induced UPR

**Fig 6:**
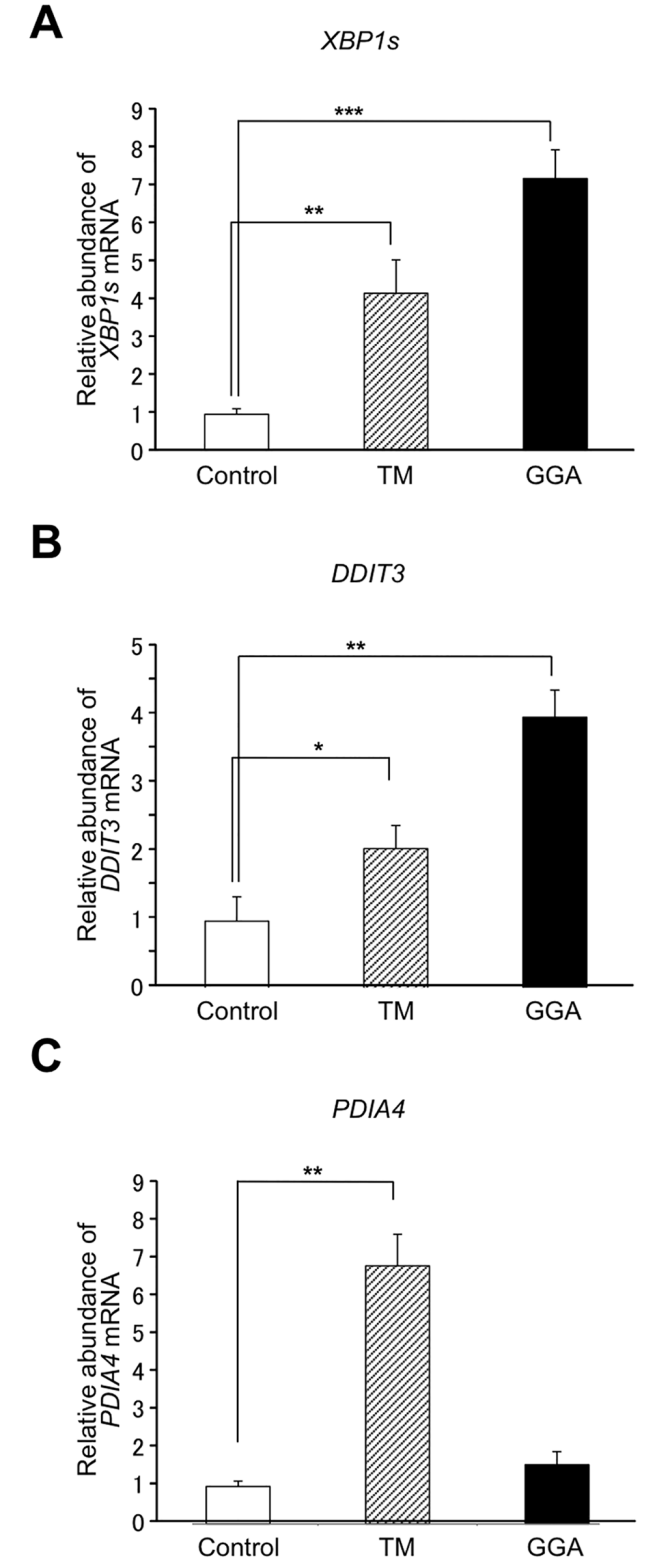
Similarity of GGA-induced UPR with palmitate-induced UPR. HuH-7 cells were treated with 20 μM GGA (**GGA**), 0.25 μg/mL tunicamycin (**TM**) or ethanol vehicle alone (**Control**) for 8 h. Then total mRNA was extracted to analyze the cellular levels of *XBP1s* (**A**) and *DDIT3* (**B**) mRNAs by RT-qPCR. HuH-7 cells were treated with 20 μM GGA (**GGA**), 0.25 μg/mL tunicamycin (**TM**) or ethanol vehicle alone (**Control**) for 24 h, and total mRNA was extracted to analyze *PDIA4* mRNA (**C**). Each point represents the mean ± SD (n = 3). Asterisks (*, **, ***) indicate statistical significance (*p* < 0.05, 0.01, 0.001, respectively) as determined by Student’s t-test.

Fig 6 demonstrates that tunicamycin treatment results in activation of the three canonical branches of UPR signaling such as *XBP1s* (mRNA processed by IRE1), *DDIT3* (PERK-downstream gene) and *PDIA4* (ATF6-target gene) in HuH-7 cells. However, GGA treatment induces only two of these, with no upregulation of *PDIA4* mRNA (Fig 6C).

**Fig 7:**
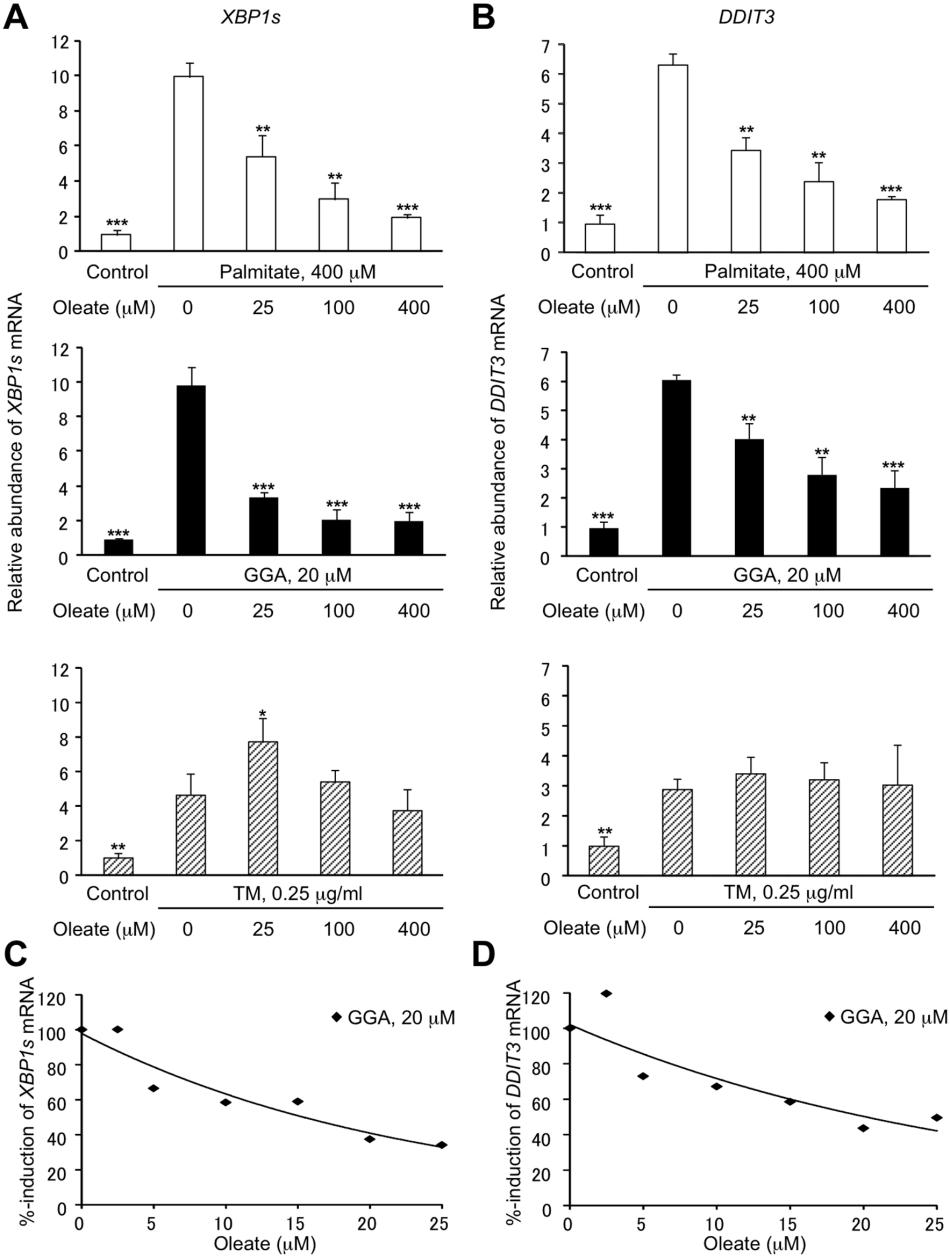
Suppression of GGA-induced UPR with oleate. HuH-7 cells were treated with 400 μM palmitate (**Palmitate**), 20 μM GGA (**GGA**) or 0.25 μg/mL of tunicamycin (**TM**) for 8 h in the presence of oleate (0, 25, 100 or 400 μM). Total mRNA was extracted to analyze the cellular levels of *XBP1s* (**A**) and *DDIT3* (**B**) mRNA by RT-qPCR. Each point represents the mean ± SD (n = 3). The asterisks (*, **, ***) indicate statistical significance (*p* < 0.05, 0.01, 0.001, respectively), compared with each relevant control induced by palmitate (400 μM), GGA (20 μM) or TM (0.25 μg/mL) alone as determined by Student’s t-test. **C**, **D**: HuH-7 cells were treated with 20 μM GGA in the absence or presence of oleate (2.5, 5, 10, 15, 20 or 25 μM) for 8 h. Total mRNA was extracted to measure the cellular levels of *XBP1s* (**C**) and *DDIT3* (**D**) mRNA by quantitative reverse-transcription (RT)-PCR in triplicate.

Lipid-induced UPR such as that triggered by palmitate is known to be suppressed by co-treatment with the mono-unsaturated fatty acid oleate [14]. Therefore, by using oleate we compared GGA-induced UPR with palmitate- induced UPR or tunicamycin-induced UPR in HuH-7 cells. As shown in top panels of Fig 7A and 7B, we were able to confirm that palmitate-induced upregulation of *XBP1s* and *DDIT3* mRNAs was dose-dependently suppressed by co-treatment with oleate in hepatoma cell lines [15]. Here we show that GGA-induced *XBP1* mRNA splicing and upregulation of *DDIT3* mRNA were both efficiently and dose-dependently suppressed by co-treatment with oleate (middle panels of Fig 7A and 7B), linear regression analysis giving IC_50_ value of 15–20 μM (Fig 7C and 7D). In contrast, tunicamycin-induced upregulation of *XBP1s* and *DDIT3* mRNAs were both unaffected by co-treatment with oleate even at its 400 μM (bottom panels of Fig 7A and 7B). As expected, oleate treatment alone upregulated neither *XBP1s* nor *DDIT3* mRNAs (data not shown).

**Fig 8:**
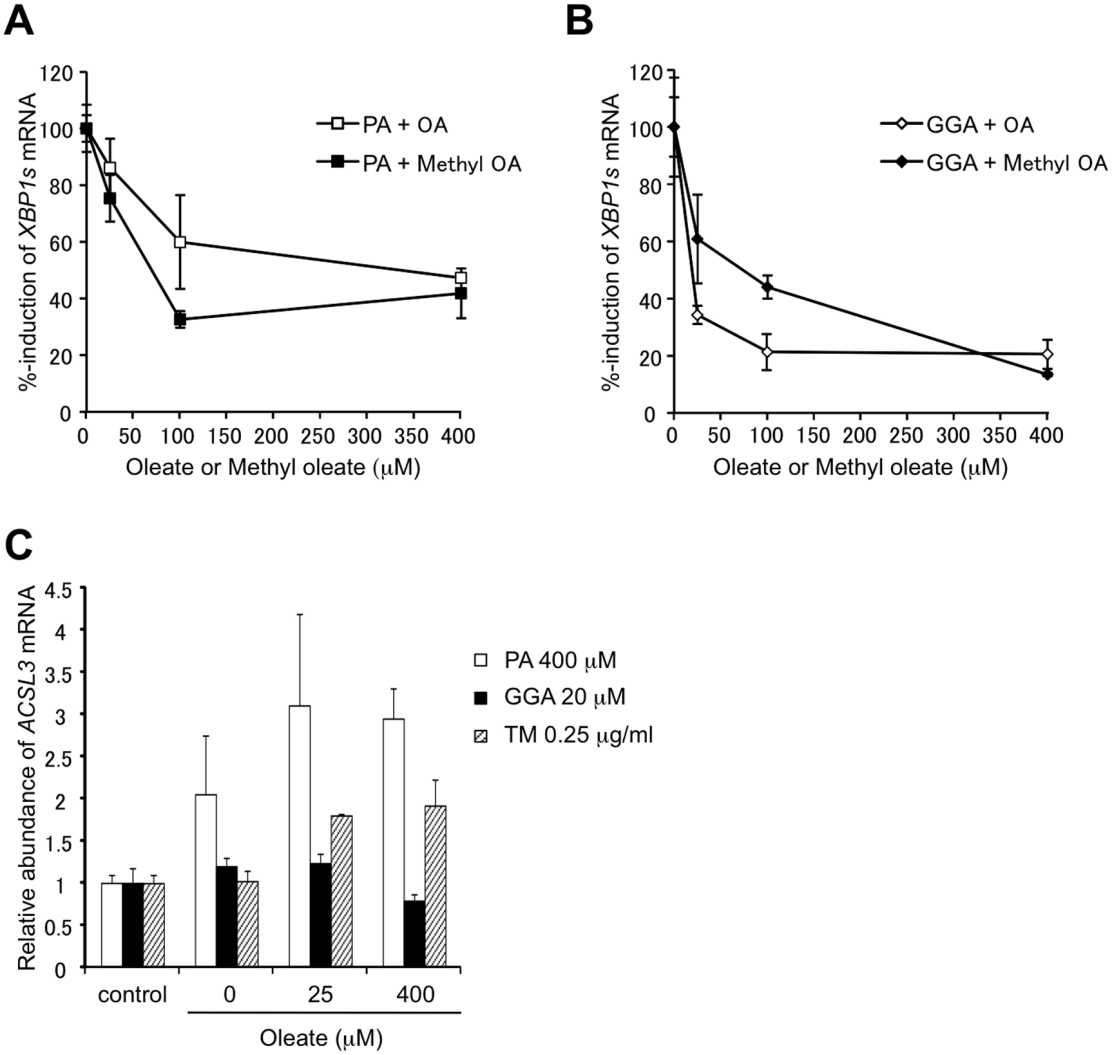
Methyl oleate-mediated suppression of GGA-induced UPR. **A**: HuH-7 cells were treated with 400 μM palmitate (PA) for 8 h in the absence or presence of 25, 100 or 400 μM oleate (open square) or methyl oleate (closed square). Total mRNA was extracted to measure the cellular level of *XBP1s* mRNA by RT-qPCR. Each point represents the mean ± SD (n = 3). **B**: HuH-7 cells were treated with 20 μM GGA for 8 h in the absence or presence of 25, 100 or 400 μM oleate (open diamond) or methyl oleate (closed diamond). Total mRNA was extracted to measure the cellular level of *XBP1s* mRNA by RT-qPCR. Each point in panel **A** and **B** represents the mean ± SD (n = 3) of % induction to palmitate (**A**) and GGA (**B**) control, respectively. The asterisks (*, **, ***) indicate statistical significance (p < 0.05, 0.01, 0.001, respectively), compared with each relevant control induced by palmitate (400 μM) or GGA (20 μM) alone as determined by Student’s t-test. **C**: HuH-7 cells were treated with vehicle alone (control), 400 μM palmitate (PA), 20 μM GGA or 0.25 μg/ml of tunicamycin (TM) for 8 h in the presence of oleate (OA) (0, 25 or 400 μM). Total mRNA was extracted to analyze the cellular levels of *ACSL3* mRNA by RT-qPCR. Each point represents the mean ± SD (n = 3).

In the literature, it has been repeatedly reported that palmitate-induced UPR is mediated by ER membrane phospholipid saturation and palmitate-induced UPR can be overcome either by membrane phospholipid unsaturation [16,17] or channeling of toxic palmitate to oleate-induced lipid droplets [18,19] with oleate overloading. In either case, overloaded oleate should be incorporated into ER membrane phospholipids or triacylglycerols in lipid droplets through oleoyl-CoA intermediate, respectively. Therefore, in place of oleate as a suppressor of GGA-induced UPR, we used oleic acid methylester or methyl oleate, which is not a direct substrate for long chain fatty acyl-CoA synthase (ACSL). Methyl oleate efficiently blocked both palmitate-induced and GGA- induced *XBP1* splicing (Fig 8A and 8B, respectively). Consistently, cellular expression of the *ACSL3* gene was not significantly induced by co-treatment with oleate (Fig 8C).

### Rescue from GGA-induced cell death by oleate co-treatment

**Fig 9:**
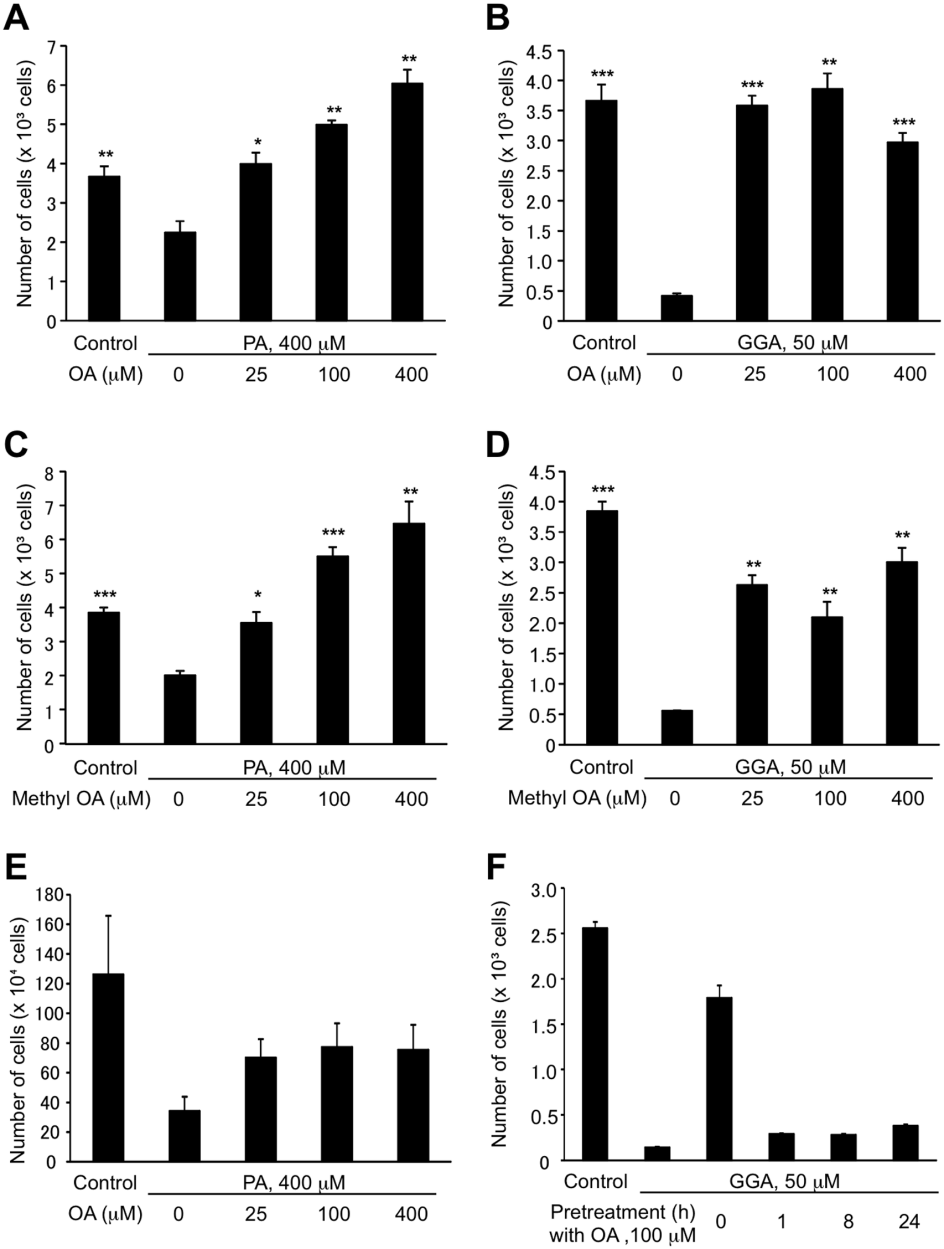
Oleate-mediated suppression of GGA-induced cell death. HuH-7 cells were treated with 400 μM palmitate (PA, **A**) or 50 μM GGA (**B**) in the absence or presence of oleate (25, 100 or 400 μM) for 24 h. The cells were treated with 400 μM palmitate (PA, **C**) or 50 μM GGA (**D**) in the absence or presence of methyl oleate (25, 100 or 400 μM) for 24 h. Viable cells were measured using the CellTiter-Glo assay kit. Values are the means ± SE (n = 3, 6 or 15). The asterisks (*, **, ***) indicate statistical significance (p < 0.05, 0.01, 0.001, respectively), compared with each relevant control induced by palmitate (400 μM) or GGA (50 μM) alone as determined by Student’s t-test. **E**: HuH-7 cells were treated with 400 μM palmitate (PA) in the absence or presence of oleate (25, 100 or 400 μM) for 24 h. Viable cells were measured using the Trypan blue method. Values are the means ± SD (n = 7). **F**: HuH-7 cells were treated with vehicle alone (control), 50 μM GGA in the absence or presence of 100 μM oleate or with pretreatment of 100 μM OA 0–24 h before 50 μM GGA treatment. 0-h pretreatment means that the cells were treated simultaneously with 50 μM GGA and 100 μM OA. Viable cells were measured using the CellTiter-Glo assay kit. Each point represents the mean± SD (n = 3, 6 or 18).

Palmitate (400 μM)- induced cell death was suppressed by 25-μM oleate co-treatment and increasing concentrations (100 and 400 μM) of oleate apparently enhanced the cell growth in the presence of 400 μM palmitate in 96-well plates measured by CellTiter Glo assay (Fig 9A). GGA at 50 μM induced complete cell death in HuH-7 cells and co-treatment with oleate even at 25 μM perfectly rescued the cells from GGA-induced cell death (Fig 9B). These preventive effects of oleate on lipid-induced cell death were mostly reproduced by methyl oleate co-treatment (Fig 9C and 9D). Suppressive effect of oleate on palmitate- induced cell death was essentially observed in 3-cm dishes also by trypan blue dye exclusion method, but the growth enhancement with higher oleate was not detected (Fig 9E).

Oleate-mediated suppression of GGA- induced cell death did not occur when the cells were pretreated with oleate 1–24 h before GGA treatment (Fig 9F).

### Inhibition of GGA-induced UPR attenuates GGA-induced accumulation of LC3β-II

**Fig 10:**
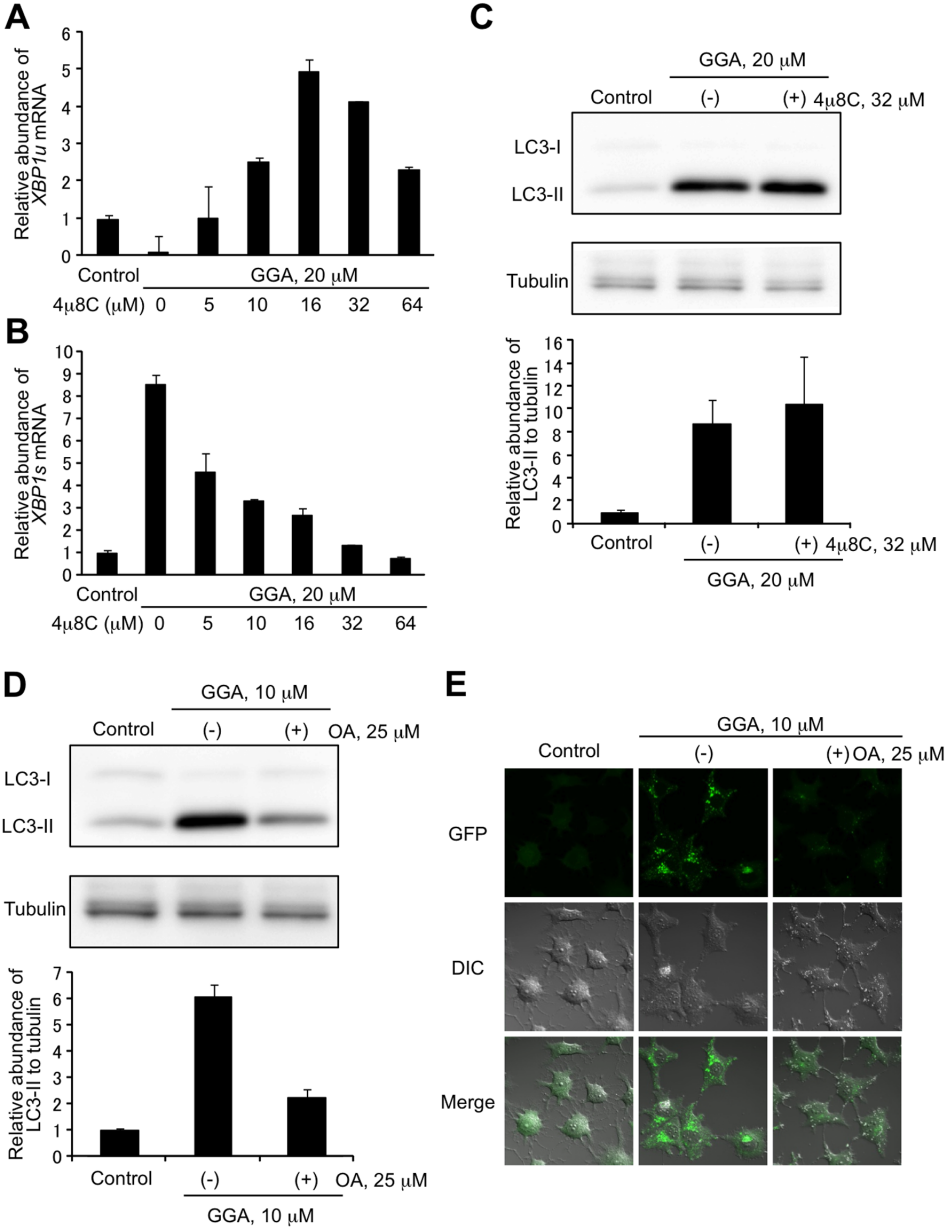
Attenuation of GGA-induced LC3β-II accumulation by oleate cotreatment. HuH-7 cells were treated with 0–64 μM 4μ8C for 8 h in the absence or presence of 20 μM GGA. Total mRNA was extracted to analyze *XBP1u* (**A**) and *XBP1s* (**B**) mRNA expression by RT-qPCR. **C**: HuH-7 cells were treated with 20 μM GGA in the absence or presence of 32 μM 4μ8C for 8 h. **D**: HuH-7 cells were treated with 10 μM GGA in the absence or presence of 25 μM oleate for 8 h. Whole-cell lysates (15 μg/lane) were prepared and LC3 levels were analyzed by western blotting. Tubulin-βIII was used as a loading control. Levels of LC3β-II expression were quantified with ImageJ densitometric analysis (mean ± SE, n = 3). Representative blots and corresponding quantification of LC3β-II / Tubulin-βIII are shown. **E**: A stable clone of HuH-7/GFP-LC3 was treated with 10 μM GGA in the absence or presence of 25 μM oleate for 8 h. Live-cell imaging was performed with the green fluorescence for GFP on an LSM700 confocal laser-scanning fluorescence microscope.

Simultaneous treatment with 4μ8C suppressed GGA-induced *XBP1* mRNA splicing in a dose-dependent manner. The presence of 5 μM 4μ8C, an inhibitor specific for IRE1 endonuclease activity, prevented GGA-induced splicing of *XBP1u* mRNA, and at higher concentrations *XBP1u* mRNA levels even increased 2-fold over the control level (Fig 10A). Consistently, 4μ8C caused a dose-dependent impairment of GGA-induced upregulation of *XBP1s* mRNA (Fig 10B). In contrast, co-treatment of HuH-7 cells with 32 μM 4μ8C did not attenuate GGA-induced accumulation of LC3β-II, a hallmark of autophagosomes, at all (Fig 10C). However, co-treatment with 25 μM oleate effectively prevented GGA-induced accumulation of LC3β-II (Fig 10D). In accordance with this biochemical finding, GGA-induced accumulation of GFP-LC3 puncta (autophagosomes) was attenuated by co-treatment with 25 μM oleate (Fig 10E).

## Discussion

We have addressed how GGA initiates signaling linked to GGA-induced cell death in human hepatoma-derived HuH-7 cells. Both GGA and 2,3-dihydroGGA immediately induce an ER stress response/UPR. Furthermore, GGA-induced *XBP1* mRNA splicing was blocked by co-treatment with oleate, indicating that GGA induces a typical lipid-induced ER stress response/UPR. GGA-induced accumulation of the autophagosome marker LC3β-II and GGA- induced cell death were also both prevented by co-treatment with oleate. Together, these data strongly demonstrate that polyunsaturated branched-chain fatty acid GGA induces ER stress response/UPR, which may be associated with GGA-induced cell death in human hepatoma cells.

GGA has been repeatedly reported to induce programmed cell death in human hepatoma cell lines [1–3,9,20]; however, the precise molecular mechanism by which this occurs remains elusive. So far, we have reported that hyperproduction of superoxide in mitochondria, increase in the cellular level of LC3β-II, dissipation of mitochondrial inner-membrane potential, and finally a massive accumulation of autophagosomes all occur following GGA treatment. Therefore, we speculated that a massive accumulation of autophagosomes or incomplete autophagic response might be directly linked to GGA-induced cell death [9]. To begin investigating this, we needed to determine exactly how GGA initiates an autophagic response. In the literature, at least three cellular conditions are known to trigger the autophagic response: starvation stress, oxidative stress, and ER stress [21]. We have previously reported that Tiron, a superoxide scavenger, slightly delayed GGA-induced conversion of LC3β-I to LC3β-II [9]. This indicates that mitochondrial oxidative stress is partially involved in triggering autophagy, but only to a small degree.

Here, we have demonstrated for the first time that GGA causes a lipid-induced ER stress-mediated UPR, as evidenced by the rapid upregulation of *XBP1* mRNA splicing and the cellular level of *DDIT3* mRNA, which may be associated with GGA-induced cell death. Of the three canonical signaling arms of the UPR, a typical lipid-induced UPR consists of IRE1 and PERK pathways [17]. *XBP1* mRNA splicing is brought about by activation of IRE1 ribonuclease activity. *XBP1s* mRNA encodes the functionally active transcription factor, whereas *XBP1u* encodes an isoform that is constitutively expressed and thought to function as a negative feedback regulator of XBP1s. Indeed, GGA induced an upregulation of cellular XBP1s levels and its nuclear accumulation by 8 h. Meanwhile, GGOH, an inactive GGA derivative that does not induce *XBP1* splicing, did not affect the subcellular distribution of XBP1s. Another branch of the lipid-induced UPR is a PERK pathway, which phosphorylates eukaryotic translation initiation factor-2α (eIF2α) and attenuates protein translation. Phosphorylation of eIF2α also allows for preferential translation of the activating transcription factor 4, which targets the *DDIT3* (formerly named as *CHOP*) gene. The rapid upregulation of PERK pathway can very well explain our previous finding of GGA-induced rapid translational attenuation of *cyclin D1* gene expression through phosphorylation of eIF2α [8].

Multiple lines of evidence have shown that lipid-induced ER stress/UPR is a possible molecular mechanism for the effects of lipotoxicity [12]. In particular, the long-chain fatty acid palmitate is well documented as an inducer of ER stress via direct sensing of the acyl-chain saturated lipid composition of the ER membrane lipid [16]. Interestingly, unlike the canonical UPR, lipid-induced ER stress does not activate the ATF6 branch of UPR [17,22]. Hence, here we have measured the cellular expression levels of the ATF6-target chaperone gene, *PDIA4* [17,23], and as a result, GGA failed to increase the cellular level of *PDIA4* mRNA (Fig 6). Furthermore, we found that GGA-induced UPR was suppressed by oleate co-treatment, as was palmitate-induced UPR. This leads us to categorize GGA-induced UPR as a form of lipid-induced ER stress or saturated fatty acid-induced ER stress, because tunicamycin-induced UPR was not attenuated by co-treatment with oleate. However, at least two points remain unclear: GGA is not a saturated fatty acid and is even a poly-unsaturated branched-chain fatty acid or 3,7,11,15- tetramethyl-2,6,10,14-hexadecatetraenoic acid, and the EC_50_ of GGA for *XBP1* splicing is under 10 μM, whereas 400–1000 μM palmitate is typically required to induce ER stress in cellular systems [15]. In any case, the present study clearly shows conclusive evidence that GGA is one of the natural lipids that induce a lipid-induced ER stress response/UPR at sub-ten μM, which is potentially responsible for lipotoxicity or lipid-induced cell death, while canonical lipid-induced ER stress response occurs with some hundreds μM saturated fatty acids such as palmitate.

Among the diterpenoids tested herein, we categorized the lipids as three groups: 1) GGA, 2,3-dihydroGGA and 9CRA induce cell death and *XBP1* splicing; 2) ATRA induces *XBP1* splicing but little cell death; and 3) phytanic acid, phytenic acid and GGOH induce neither cell death nor *XBP1* splicing. In the near future experiment, we should clarify why ATRA-induced UPR is not linked to cell death, whereas GGA-induced UPR is apparently linked to cell death in HuH-7 cells.

Scant evidence for a molecular role of ER stress response/UPR in triggering autophagy is currently available. A single report of XBP1s directly binding the beclin 1 (*BECN1*) gene promoter exists [24]. These authors concluded that *XBP1* splicing triggers an autophagic signal pathway through XBP1s-mediated transcriptional regulation of the *BECN1* gene, which product consists a triggering complex of autophagy with PI3-kinase [24]. Therefore, we assessed the effect of a specific IRE1 endonuclease inhibitor, 4μ8C, on GGA-induced autophagy. This inhibitor completely blocked GGA-induced *XBP1* splicing but did not attenuated GGA-induced cellular accumulation of LC3β-II, suggesting that *XBP1* splicing may not be an upstream signal for the GGA-induced incomplete autophagic response.

Rather than co-treatment with 4μ8C, co-treatment with oleate more broadly prevents GGA-induced UPR including IRE1 and PERK pathways. The mono-unsaturated fatty acid attenuated GGA-induced accumulation of autophagosomes to the greater extent (Fig 10E), suggesting that except *XBP1* splicing, some other GGA-inducible UPRs such as IRE1 kinase cascade and PERK pathway may cause a shift in cellular autophagic response [25]. On the other hand, we cannot exclude the possibility that incomplete autophagic response induced by GGA may be the signal upstream of the UPR [26–28].

In conclusion, we describe here that GGA induces ER stress/UPR, which may be associated with GGA-induced cell death. We have further demonstrated that GGA-induced UPR could be an upstream signal for the GGA-induced incomplete autophagic response.

## Supporting Information

S1 File S1 Table. The nucleotide sequences of each primers used for real-time RT-PCR. S2 Table. The condition of thermal cycler for real-time RT-PCR of *XBP1u*. S3 Table. The condition of thermal cycler for real-time RT-PCR of *XBP1s*. S4 Table. The condition of thermal cycler for real-time RT-PCR of *DDIT3*. S5 Table. The condition of thermal cycler for real-time RT-PCR of *PDIA4*. S6 Table. The condition of thermal cycler for real-time RT-PCR of *ACSL3*. S7 Table. The condition of thermal cycler for real-time RT-PCR of *28S rRNA*.
(DOC)
