# Supplementary material for: Polyunsaturated Branched-Chain Fatty Acid Geranylgeranoic Acid Induces Unfolded Protein Response in Human Hepatoma Cells
Source: PLoS One. 2015 Jul 17;10(7):e0132761. doi: 10.1371/journal.pone.0132761 (PMC4506074; doi:10.1371/journal.pone.0132761)
Supplement: S1 File — S1 Table. The nucleotide sequences of each primers used for real-time RT-PCR. S2 Table. The condition of thermal cycler for real-time RT-PCR of XBP1u. S3 Table. The condition of thermal cycler for real-time RT-PCR of XBP1s. S4 Table. The condition of thermal cycler for real-time RT-PCR of DDIT3. S5 Table. The condition of thermal cycler for real-time RT-PCR of PDIA4. S6 Table. The condition of thermal cycler for real-time RT-PCR of ACSL3. S7 Table. The condition of thermal cycler for real-time RT-PCR of 28S rRNA. (DOC) [file pone.0132761.s001.doc]

S1 Table. The nucleotide sequences of each primers used for real-time RT-PCR.

| Genes | Primer | Sequence (5’ – 3’) |
| --- | --- | --- |
| *DDIT3* | F | ATGGCAGCTGAGTCATTGCCTTTC |
|  | R | AGAAGCAGGGTCAAGAGTGGTGAA |
| *PDIA4* | F | CGCGAGTTTGTCACTGCTTTC |
|  | R | CGTCCTTCTTGGGGTCCATC |
| *ACSL3* | F | CAAGGGCATCATTGTGCAT |
|  | R | GGGCAATGGTTTGCTATGAG |
| *28S rRNA* | F | TTAGTGACGCGCATGAATGG |
|  | R | TGTGGTTTCGCTGGATAGTAGGT |

F: forward primer, R: reverse primer

S2 Table. The condition of thermal cycler for real-time RT-PCR of *XBP1u*.

| ***XBP1u*** | Temperature, duration | Slope |
| --- | --- | --- |
| Denature | 95ºC, 600 s | 20ºC / s |
| PCR (45 cycles) | 95ºC, 10 s | 20ºC / s |
|  | 65ºC, 20 s | 20ºC / s |
|  | 72ºC, 20 s | 20ºC / s |
| Melting | 95ºC, 0 s | 20ºC / s |
|  | 57ºC, 15 s | 20ºC / s |
|  | 98ºC, 0 s | 0.1ºC / s |
| Cooling | 40ºC, 30 s | 20ºC / s |

S3 Table. The condition of thermal cycler for real-time RT-PCR of *XBP1s*.

| ***XBP1s*** | Temperature, duration | Slope |
| --- | --- | --- |
| Denature | 95ºC, 600 s | 20ºC / s |
| PCR (45 cycles) | 95ºC, 10 s | 20ºC / s |
|  | 62ºC, 20 s | 20ºC / s |
|  | 72ºC, 20 s | 20ºC / s |
| Melting | 95ºC, 0 s | 20ºC / s |
|  | 57ºC, 15 s | 20ºC / s |
|  | 98ºC, 0 s | 0.1ºC / s |
| Cooling | 40ºC, 30 s | 20ºC / s |

S4 Table. The condition of thermal cycler for real-time RT-PCR of *DDIT3*.

| ***DDIT3*** | Temperature, duration | Slope |
| --- | --- | --- |
| Denature | 95ºC, 600 s | 20ºC / s |
| PCR (40 cycles) | 95ºC, 10 s | 20ºC / s |
|  | 54ºC, 20 s | 20ºC / s |
|  | 72ºC, 20 s | 20ºC / s |
| Melting | 95ºC, 0 s | 20ºC / s |
|  | 57ºC, 15 s | 20ºC / s |
|  | 98ºC, 0 s | 0.1ºC / s |
| Cooling | 40ºC, 30 s | 20ºC / s |

S5 Table. The condition of thermal cycler for real-time RT-PCR of *PDIA4*.

| ***PDIA4*** | Temperature, Duration | Slope |
| --- | --- | --- |
| Denature | 95ºC, 600 s | 20ºC / s |
| PCR (40 cycles) | 95ºC, 10 s | 20ºC / s |
|  | 59ºC, 20 s | 20ºC / s |
|  | 72ºC, 20 s | 20ºC / s |
| Melting | 95ºC, 0 s | 20ºC / s |
|  | 57ºC, 15 s | 20ºC / s |
|  | 98ºC, 0 s | 0.1ºC / s |
| Cooling | 40ºC, 30 s | 20ºC / s |

S6 Table. The condition of thermal cycler for real-time RT-PCR of *ACSL3*.

| ***ACSL3*** | Temperature, Duration | Slope |
| --- | --- | --- |
| Denature | 95ºC, 600 s | 20ºC / s |
| PCR (42 cycles) | 95ºC, 15 s | 20ºC / s |
|  | 60ºC, 60 s | 20ºC / s |
| Melting | 95ºC, 0 s | 20ºC / s |
|  | 57ºC, 15 s | 20ºC / s |
|  | 98ºC, 0 s | 0.1ºC / s |
| Cooling | 40ºC, 30 s | 20ºC / s |

S7 Table. The condition of thermal cycler for real-time RT-PCR of *28S rRNA*.

| ***28S rRNA*** | Temperature, Duration | Slope |
| --- | --- | --- |
| Denature | 95ºC, 600 s | 20ºC / s |
| PCR (40 cycles) | 95ºC, 15 s | 20ºC / s |
|  | 60ºC, 30 s | 20ºC / s |
| Melting | 95ºC, 0 s | 20ºC / s |
|  | 65ºC, 15 s | 20ºC / s |
|  | 95ºC, 0 s | 0.1ºC / s |
| Cooling | 40ºC, 30 s | 20ºC / s |
